# Targeted cancer therapy with ribosome biogenesis inhibitors: a real possibility?

**DOI:** 10.18632/oncotarget.5775

**Published:** 2015-09-22

**Authors:** Elisa Brighenti, Davide Treré, Massimo Derenzini

**Affiliations:** ^1^ Department of Experimental, Diagnostic and Specialty Medicine, Bologna University, Bologna, Italy

**Keywords:** cancer chemotherapy, ribosome biogenesis inhibitors, nucleolus, cell cycle, p53

## Abstract

The effects of many chemotherapeutic drugs on ribosome biogenesis have been underestimated for a long time. Indeed, many drugs currently used for cancer treatment – and which are known to either damage DNA or hinder DNA synthesis – have been shown to exert their toxic action mainly by inhibiting rRNA synthesis or maturation. Moreover, there are new drugs that have been proposed recently for cancer chemotherapy, which only hinder ribosome biogenesis without any genotoxic activity. Even though ribosome biogenesis occurs in both normal and cancer cells, whether resting or proliferating, there is evidence that the selective inhibition of ribosome biogenesis may, in some instances, result in a selective damage to neoplastic cells. The higher sensitivity of cancer cells to inhibitors of rRNA synthesis appears to be the consequence of either the loss of the mechanisms controlling the cell cycle progression or the acquisition of activating oncogene and inactivating tumor suppressor gene mutations that up-regulate the ribosome biogenesis rate. This article reviews those cancer cell characteristics on which the selective cancer cell cytotoxicity induced by the inhibitors of ribosome biogenesis is based.

## INTRODUCTION

Many drugs used for treating cancer, such as DNA-reactive agents, antimetabolites, and topoisomerase inhibitors, exert their toxic action by damaging DNA or hindering DNA synthesis. The rationale for this chemotherapeutic approach is that DNA integrity and duplication are crucial for proper cellular function and proliferation, respectively. In proliferating normal cells, the damage or inhibition of DNA is sensed by cell-cycle checkpoint factors that block cell cycle progression, thus making it possible for the cell to repair DNA before division (see for review [1-3]). The repair of these lesions is important in preventing apoptotic cell death. In proliferating cancer cells these mechanisms frequently function poorly or not at all [4, 5], so DNA damages may more often induce cell death [6]. Therefore, these chemotherapeutic agents may be considered to be more effective against cancer cells than normal proliferating cells. On the other hand, these kinds of drugs, apart from their action on DNA, very frequently also induce an inhibition of ribosome biogenesis [7]. This fact would appear to reduce the specificity of these drugs for cancer cell elimination. In fact, unlike DNA synthesis, the synthesis of rRNA occurs in both proliferating and resting cells, the latter constituting a large portion of normal tissues. However, a series of recent results indicated that - in some instances - a specific, non-genotoxic inhibition of rRNA transcription may result in a selective damage to neoplastic cells (reviewed in [8-12]). Data dealing with the alterations in the relationship between ribosome biogenesis and cell proliferation, as well as with the changes in the mechanisms controlling the ribosome biogenesis rate in cancer cells, may explain the selective cytotoxicity of ribosome biogenesis inhibitors for cancer cells [13-17]. These characteristics - which may be of importance for the selection of an appropriate anticancer therapy on the one hand, and the stimulation of the development of specific rRNA inhibitors on the other - are the subject of this review. For an easier understanding of the topics discussed, a brief description of the main steps in ribosome biogenesis and of its relationship with cell proliferation will be given first.

### Ribosome biogenesis and cell proliferation

Ribosome biogenesis is the result of a series of coordinated steps that occur in the nucleolus (reviewed in [18-21]). Within the nucleolus, some ribosomal genes are transcribed by RNA polymerase I (Pol I) to produce the 47S rRNA precursor that is then processed in order to generate the mature 18S, 5.8S, and 28S rRNA. The 5S rRNA, which is transcribed in the nucleoplasm by RNA Polymerase III (Pol III), is imported to the nucleolus. The assembly of a specific multiprotein complex at the rDNA promoter containing Pol I is necessary for the initiation of 47S pre-rRNA synthesis. Within this multiprotein complex, at least three basal factors - the ribosomal DNA transcription factor Rrn3 [22] (also referred to as Transcription Initiation Factor I (TIF-I) A [23]), Selectivity factor 1 (SL1), and Upstream Binding Factor (UBF) - are necessary for ribosome gene transcription in mammals [24].

TFIIIC and TFIIIB transcription factors are necessary for the transcription of the 5S rRNA by Pol III [25-27]. The ribosomal proteins (RPs), whose mRNA is transcribed by RNA Polymerase II (Pol II), are also imported to the nucleolus where they assemble with the rRNAs to form both the large pre-60S and the small pre-40S incompletely processed subunits of the final mature ribosomal subunits. The large 60S subunit contains one each of the 28S, 5.8S, and 5S RNAs, together with 47 ribosomal proteins, called RPLs, whereas the small 40S subunit contains only the 18S RNA and 32 ribosomal proteins, called RPSs [28, 29]. The large and small subunits migrate to the cytoplasm, where they make up the final 80S ribosome particle. In proliferating cells, the ribosome biogenesis rate appears to be regulated by cell proliferation-controlling processes [30]. During mitosis, Pol I transcription is repressed by the CDK1-cyclin B kinase activity, and re-activation of Pol I transcription at the end of mitosis depends on inhibition of this activity [31-34]. The RNA-polymerase I upstream binding factor (UBF), inactive during mitosis and early G1 phase [35], is phosphorylated by G1-specific cyclin/Cdk complexes thus stimulating rRNA synthesis during G1 phase progression [36]. Phosphorylation of the transcription factor Rrn3/TIF-IA and of SL1 also correlate with cell cycle fluctuation of rDNA transcription [24, 37, 38]. Moreover, in cycling cells, the phosphorylation of the pRb tumor suppressor - induced by the cyclin-D-cyclin-dependent protein kinase (CDK)-4, CDK-6, and cyclin E-CDK-2 complexes during the G1 phase - hinders its binding to UBF and TFIIIB, thus allowing rRNA transcription to increase. In fact, pRb, in its active, non-phosphorylated form, inhibits both rRNA synthesis by binding to UBF [39-42] and Pol III transcription by binding to TFIIIB [43, 44]. Indeed, an up-regulation of the rate of ribosome biogenesis is necessary for the enhanced protein synthesis requested by cells in order to grow in size during the cell cycle phases, thus consequently ensuring the generation of normal-sized, viable daughter cells [45]. In this context, it was demonstrated that not so much the capacity of protein synthesis, but rather the production of new ribosomes is important for cell cycle progression [46], a deficiency in ribosome biogenesis activating in fact a p53-dependent checkpoint mechanism [47, 48]. Furthermore, the relationship between ribosome biogenesis rate and cell cycle progression was stressed by the finding that an accelerated or delayed achievement of the appropriate amount of ribosomes during the G1 phase is associated with an accelerated or delayed G1/S-phase progression [49].

### The inhibitors of rRNA synthesis induce apoptotic death in cancer cells lacking the p53-pRb control of G1/S phase transition

**Figure 1:**
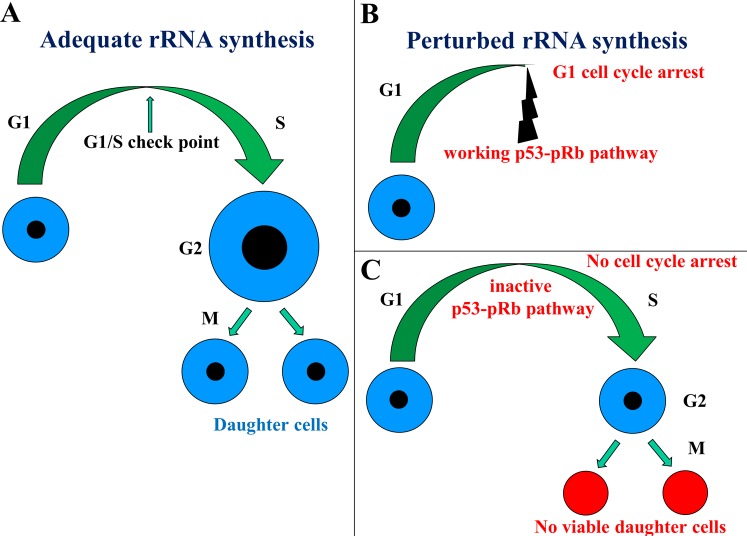
Schematic representation of the relationship between cell cycle progression and ribosome biogenesis When ribosome biogenesis is adequate, the cell increases in size, passes through the cell cycle phases, and gives rise to two normal daughter cells **A.** In the case of perturbed ribosome biogenesis, if the p53-pRb pathway normally controls the G1/S phase checkpoint, the cell cycle progression is arrested in the G1 phase **B.** However, if the p53-pRb pathway is not functioning, the cell cycle progression is not arrested and the cell divides without having reached a sufficient size, thus giving rise to two non-viable daughter cells **C.**

During cell cycle progression, there are some active mechanisms that ensure the proper timing of cell cycle events by enforcing the dependence of late events on the completion of early events [50]. These checkpoints exert their function at the G1-S and G2-M phase transitions by arresting cells which, for any reason, should not enter the following phase. Inappropriate ribosome biogenesis appears to be one of these reasons, with perturbed rRNA processing and ribosome assembly inducing cell cycle arrest in a p53-dependent manner [47, 51-56]. There is evidence that any perturbation in ribosome biogenesis causes p53 accumulation and activation. Indeed, in normal conditions, the amount of p53 within the cell is very small due to the fact that p53 is a short-lived protein that is rapidly degraded by MDM2 (Murine Double Minute 2) and HDM2 in humans, which acts as an E3 ubiquitin ligase facilitating p53 proteasomal degradation. Ribosome biogenesis perturbation is responsible for the fact that several ribosomal proteins (RPs), no longer used for ribosome construction, may bind to MDM2, thus relieving its inhibitory activity toward p53 which, therefore, accumulates within the cell nucleus (reviewed in [57, 58]). The most important RPs for the inactivation of MDM2 are RPL5 and RPL11 [55, 59-61], which, by forming a complex with the 5S rRNA bind to and inactivate MDM2, all the components of the complex being necessary for the its inhibitory function [62, 63]. Ribosome biogenesis perturbations block the transition from G1 to S-phase. The mechanism involved in the block of cell cycle progression is activated by p53 that induces p21 expression, which in turn - by hindering pRb phosphorylation - blocks the activity of E2Fs transcription regulators and the consequent transit from G1 to S phase [2, 64]. Thus the activation of the p53-pRb pathway for blocking cell cycle progression in conditions of insufficient ribosome biogenesis is an important mechanism for preventing proliferating cells from dividing without reaching an outfit of the cell constituents sufficient for daughter cell survival. Cancer cells are frequently characterized by i) the presence of activating mutations of gene coding for the components of the proliferating and growth factor signaling pathways, ii) the disruption of pRb function consequent to RB1 mutation or deletion, overexpression of cyclin D1, cdk4, and cyclin E, and INK4a mutation, gene deletion, or silencing [65, 66], and iii) inactivating mutations of p53 [67, 68]. A frequent effect of these changes is the loss of a normal G1/S phase checkpoint, with the consequent loss of the functional relationship between ribosome biogenesis and cell cycle progression [13, 14]. What were the consequences of the absence of an efficient G1/S phase checkpoint in proliferating cells upon ribosome biogenesis perturbation? Experimental evidence indicates that, in cells with a normally functioning p53-pRb pathway, the specific inhibition of ribosome biogenesis induced by Actinomycin D (ActD) treatment at a dose that selectively blocks the Pol I-dependent transcription caused an arrest in cell cycle progression that hindered the cell division with an incomplete ribosome content [13]. When rRNA synthesis was resumed, ribosome biogenesis tended to continue to completion and the cell divided with an appropriate ribosome content, as demonstrated by the absence of quantitative changes in the rRNA content in these cells, even after a 1 h treatment with ActD every day for four days. In contrast, in the case of cells lacking both p53 and pRb, the exposure to ActD did not influence the cell cycle progression for the absence of the activation of the cell-cycle check-points [13], thus leading these cells to divide without having reached an appropriate ribosome content; therefore, the progressive reduction in the ribosome content becomes very quickly incompatible with cell life (Figure 1). In fact, in this case, after ActD treatment a progressively increased cell death rate occurred, due to an increased apoptotic activity, without significant changes in the cell cycle progression rate [13]. The importance of the status of the p53-pRb-mediated control of cell cycle progression in the cell response to chemotherapeutic agents that hinder ribosome biogenesis was also suggested by a study on the effects of 5-Fluorouracyl (5-FU) and methotrexate treatment on human cancer cell lines, after silencing for RB1 expression. In fact, besides the effect on DNA synthesis, a major effect of both drugs is the down-regulation of ribosome biogenesis: 5-FU by inhibiting rRNA processing, and methotrexate by inhibiting rRNA transcription [7]. Therefore, the inhibition of ribosome biogenesis can be considered for a good part responsible for the effects caused by these two drugs in cancer cells. Treatments with 5-FU and methotrexate, at doses and time exposures derived from the evaluation of the interstitial pharmacokinetics of the drugs in vivo [69], caused a marked reduction in the RB1-silenced cancer cell population growth, but not in control cells. Moreover, a higher death rate was observed in drug-treated RB1-silenced cells than in control cells [14]. Therefore, the deficiency of the pRb and p53 function, which appeared to give cells an immediate advantage in maintaining proliferation unchanged after ribosome biogenesis inhibition, was on the contrary responsible for the later cell population exhaustion (Figure 1). These observations taken together indicate that the absence of the p53-pRb mediated check-point control, as it occur in many cancer cells, may render them more sensitive to the inhibition of ribosome biogenesis than normal cells. In other words, the loss of the two major tumor suppressors is a cancer characteristic that should be exploited in order to selectively eliminate cancer cells by using inhibitors of ribosome biogenesis. In support of this statement are some clinical findings indicating that patients with breast cancers lacking a functioning p53/pRb pathway and treated with the adjuvant standard chemotherapy regimen, which includes 5-FU and methotrexate, had a more favorable clinical outcome in comparison with patients with cancers with a normally functioning p53/pRb pathway [14, 15].

### Ribosome biogenesis has a highly variable rate in cancer cells

**Figure 2:**
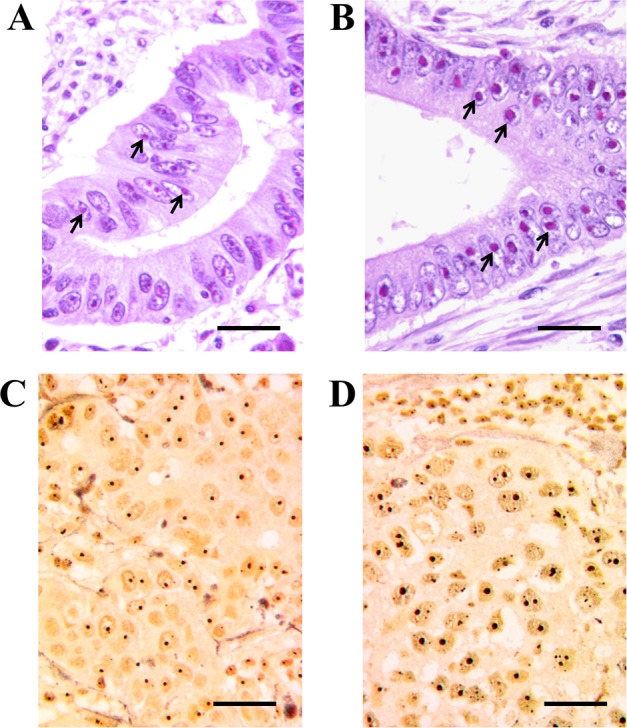
High variability of the nucleolar size in human cancers **A.** and **B.** Histological sections from two routinely processed colon adenocarcinomas stained with hematoxylin and eosin (H&E). The nucleolus frequently appears as a roundish body mainly stained with eosin, due to its high protein content. Compare the small size of the nucleoli in cancer sample **A.** with the very enlarged nucleoli in cancer sample **B.** Both cancers were p53 wild-type. Arrows indicate nucleoli. Bar, 25 μm. **C.** and **D.** Histological sections from two routinely processed infiltrating ductal breast cancers, specifically silver-stained for the argyrophilic nucleolar proteins. Both cancers were p53 wild-type. Nucleoli are very darkly stained by silver. The size of nucleoli in breast cancer sample **C.** is very small in comparison with that of nucleoli in breast cancer sample **D.** Bar, 25 μm.

It is a common belief that cancer cells are typically characterized by a higher rate of ribosome biogenesis than the corresponding normal cells. An up-regulated ribosome biogenesis in cancer cells may be considered to be the consequence of the fact that neoplastic transformation is frequently characterized by changes of proto-oncogenes and tumor suppressor genes [5] which activate mechanisms stimulating cell growth and proliferation, and also trigger a series of pathways which enhance ribosome biogenesis [70]. The extracellular signal-regulated kinase (MAPK/ERK) pathway activates both Pol I transcription, through the phosphorylation of UBF [71, 72], and Pol III transcription, by phosphorylating TFIIIB [21], and ERK phosphorylates the Transcription Initiation Factor TIF-IA which links the initiation-competent Pol I entity with the rDNA promoter [38]. Mitogens and growth factors also activate the PI3K/AKT pathway, which in turn activates MYC [73], the major modulator of ribosome biogenesis. MYC increases Pol I activity by enhancing the recruitment of SL1 to promoters, stimulates ribosomal protein synthesis by increasing Pol II transcription, and facilitates Pol III transcription by activating TFIIIB [27, 74, 75]. Furthermore, the mitogenic growth factor stimulation, through the activation of the mammalian target of rapamycin (mTOR), also induces both Pol I transcription by activating UBF and Rrn3/TIF-IA (the latter not in all cell types), and Pol III transcription by facilitating the association of the transcription factors TFIIIB and TFIIIC with 5S rRNA genes [18, 76]. Furthermore, there is evidence that the products of the tumor suppressor genes that adversely affect cell proliferation and cell cycle progression also negatively control ribosome biogenesis. In fact, p53 inhibits both Pol I transcription by binding to the selectivity factor SL1 - which is necessary for Pol I recruitment to the rRNA gene promoter [77] - and Pol III transcription by binding to TFIIIB [44]. In the control of ribosome biogenesis, p53 may be aided by p14ARF. In addition to activating the p53 pathway, this tumor suppressor hinders ribosome biogenesis both by inhibiting UBF recruitment on the transcription complex [78], and by down-regulating the activity of nucleophosmin, a multifunctional nucleolar protein involved in rRNA processing [79]. As for the other major tumor suppressor, pRb, it inhibits ribosome biogenesis, as reported above [39-44]. Lastly, Pol I transcription is also repressed by PTEN (phosphatase and tensin homolog deleted in chromosome 10), another important tumor suppressor which activates various signaling events that inhibit cell proliferation and disrupt the SL1 complex [80]. Therefore, the claim that a high activity of ribosome biogenesis characterizes cancer cells appears to be substantiated by the observation that many of the genetic changes occurring in cancer cells up-regulate ribosome biogenesis. On the other hand, there is evidence that the severity of these changes may be highly variable in human cancers, thus giving rise to tumors characterized by a highly variable ribosome biogenesis rate, which is sometimes quite similar to that of the corresponding normal cells [81]. The highly variability of the nucleolar size in cancer cells is long since known [82, 83]. Of importance from the histopathological standpoint is the fact that the variability in the rate of ribosome biogenesis is reflected in differently-sized nucleoli within cancer cells, the size of the nucleolus being directly related to the rate of ribosome biogenesis [84, 85]. Accordingly, nucleoli with highly variable size can be observed in histological sections in the same types of cancers, either stained with E&E (Figures 2A and 2B) or with the silver staining procedure selective for nucleolar visualization (Figures 2C and 2D) (reviewed in [86-88]).

### The inhibitors of rRNA synthesis induce apoptotic death in cancer cells with up-regulated ribosome biogenesis

**Figure 3:**
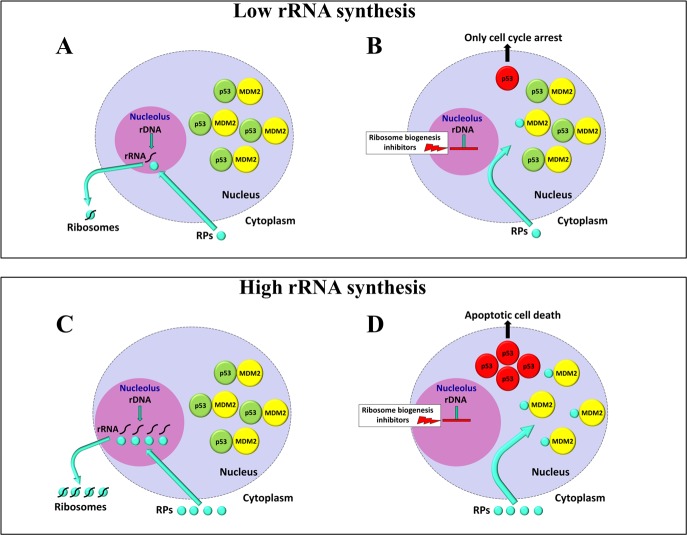
Schematic representation of the effects of ribosome biogenesis inhibitors on cells with different rates of rRNA synthesis Normally, the nuclear p53 level is very low as a consequence of the fact that newly synthesized p53 is rapidly linked by the oncoprotein MDM2 which mediates the tumour suppressor ubiquitylation and proteasome-dependent degradation. In cells with a low ribosome biogenesis rate, also a low production of ribosomal proteins (RPs) occurs **A.** In these cells, after the inhibition of ribosome biogenesis, just a few RPs, no longer used for ribosome building, bind to a very few MDM2 molecules, thus only partially neutralizing their ubiquitin ligase activity toward p53, with a consequent induction of a low-level p53 stabilization. A low amount of stabilized p53 is responsible for cell cycle arrest **B.** In the case of cells characterized by a high ribosome biogenesis rate also a high production of RPs occurs **C.** The inhibition of rRNA synthesis causes a large number of RPs, no longer used for ribosome building, to bind and neutralize a large number of MDM2 molecules, thus inducing a strong p53 stabilization. A high amount of stabilized p53 is responsible for cell apoptotic death **D.**

This high variability in the ribosome biogenesis rate in cancer cells has been found to account for a different sensitivity of cancer cells with functional p53 to the treatment with inhibitors of rRNA synthesis. Recent data indicate that cancer cells exhibiting an up-regulated ribosome biogenesis are highly sensitive to drugs inhibiting rRNA transcription or maturation [17]. In fact, it has been shown that the exposure of human cancer cell lines characterized by different levels of rRNA transcription to drugs which inhibit rRNA synthesis induced apoptosis only in the cells with the highest rates of ribosome biogenesis. Moreover, the cancer cells in which the high rRNA synthesis was down-regulated by serum starvation failed to undergo apoptosis after the treatment with rRNA synthesis inhibitors. The induction of apoptosis by ribosome biogenesis inhibitors in cells with high but not with low rRNA synthesis rate was due to the fact that the level of p53 stabilization and of its activity in the activation of the target genes responsible for apoptosis induction were directly related to the rRNA synthesis rate of the cells before the drug treatment. Apoptotic cell death is induced only in those cells where a high amount of p53 is stabilized after rRNA synthesis inhibition. This is consistent with previously reported data on the relationship between p53 levels and the induction of apoptosis [89]. Interestingly, the inhibition of rRNA synthesis always stopped the cell cycle, irrespective of the ribosome biogenesis rate of cells. The high and low levels of p53 stabilization induced by rRNA synthesis inhibitors were the consequence of the fact that high and low amounts of ribosomal proteins, no longer used for ribosome building, bind to the ubiquitin ligase MDM2, thus hindering p53 ubiquitylation and proteasomal degradation (Figure 3), according to the well-established RP-MDM2 pathway that controls the cellular level of p53 (see reviews in [57, 58]). The level of p53 stabilization induced by drugs acting in different ways from the inhibition of ribosome biogenesis, such as Hydroxyurea [90], was independent of the level of ribosome biogenesis in cells and lower than that occurring after the inhibition of rRNA synthesis. Worth of note, in cells with a low ribosome biogenesis rate, the combined treatment with Actinomycin D and Hydroxyurea exerted an additive effect on p53 stabilization, thus succeeding in the apoptotic pathway activation even in these cells. The different sensitivity to inhibition of rRNA synthesis depending on a different rate of ribosome biogenesis is very likely at the basis of the preferential induction of apoptosis in tumor cells - when compared to the normal cells of the same lineage - by the selective rRNA synthesis inhibitor CX-5461 [10, 91]. CX-5461, a non-genotoxic drug recently undergoing phase I clinical trials for the treatment of hematologic malignancies, inhibits ribosome biogenesis, most likely by disrupting the SL-1/rDNA complex [92, 93]. It has been shown that CX-5461 induced p53-dependent apoptosis of malignant B cells, but not of normal cells, in a Eμ-MYC mouse model of Burkitt lymphoma, resulting in an increased survival rate of tumor-bearing mice [91]. In these mice, B-lymphocytes constitutively overexpressing MYC are characterized by an enhanced ribosome biogenesis, due to an increased rDNA transcription rates and Pol I machinery abundance [91]. Indeed, MYC controls ribosome biogenesis by stimulating the synthesis of all three DNA-dependent RNA polymerases, thus enhancing the synthesis of 47S pre-rRNA, 5S rRNA and ribosomal proteins, which are necessary for ribosome building [94]. That the high sensitivity to CX-5461 was the consequence of the activation of p53 was demonstrated by the fact that Eμ-MYC lymphoma cells, with elevated basal rates of Pol I transcription, mutant or null for p53 exhibited a 180-fold decreased sensitivity to the drug [91]. On the other hand, the lack of cytotoxic effects in normal cells without up-regulated Pol-1 transcription suggested that the rate of rRNA synthesis of the cells could account for the different sensitivity of cancer cells with functional p53 to the treatment with inhibitors of rRNA synthesis. Therefore, in tumor cells with up-regulated ribosome biogenesis consequent to MYC overexpression, the inhibition of rDNA transcription may well be responsible for a high accumulation and activation of p53, sufficient for the induction of apoptosis, whereas it causes only transient effects in cells with a normally regulated ribosome biogenesis (Figure 3).

### Future perspectives and conclusions

The effect of many chemotherapeutic drugs on ribosome biogenesis has been underestimated for a long time. Only recently, it has been shown that the mechanism of action of many drugs used for cancer treatment is mainly based on either the inhibition of rRNA synthesis or maturation [7]. The introduction in cancer chemotherapy of CX-5461, a molecule which selectively hinders Pol I activity without exerting any genotoxic activity, may certainly stimulate more studies aiming to identify and/or produce compounds that have these characteristics for targeting the nucleolus in cancer cells [95]. In this context, worthy of mention are the recent results obtained using a small molecular compound, BMH-21, and a small-molecule peptide (22mer) which have been found to stabilize p53 by inhibiting rDNA transcription. BMH-21 binds to GC-rich sequences and inhibits RNA Pol I activity [96]. It also induces the proteasome-dependent destruction of the large catalytic subunit in the Pol I complex, as do three other small molecular compounds, BMH-9, BMH-22, and BMH-23 [97]. The 22mer targets the interface between RNA polymerase I and Rrn3 thus selectively inhibiting the synthesis of rRNA [98]. Indeed, the development of similar compounds appears to be particularly appropriate, based on the evidence that cancer cells may acquire genetic and metabolic changes that render them much more sensitive to the inhibition of rRNA synthesis than normal cells. Considering the fact that these changes consist of either the loss of the mechanisms controlling the relationship between cell growth and cell cycle progression or an up-regulated ribosome biogenesis, it could be rationally suggested that a pre-treatment analysis should be conducted on cancer samples to define the integrity of their mechanism regulating the G1/S phase checkpoint and to evaluate the rate of ribosome biogenesis. Such a characterization can be carried out very easily and should be very useful for distinguishing those cancers that may benefit greatly from treatment with ribosome biogenesis inhibitors, resulting in apoptosis, from those in which the inhibition of ribosome biogenesis will cause only a cell cycle arrest with a low chemotherapeutic efficacy. A schematic representation of the effects of treatment with inhibitors of ribosome biogenesis in relation to the characteristics of neoplastic cells is shown in Table 1.

**Table 1:** Summary diagram of the different efficacy of treatments with inhibitors of ribosome biogenesis in relation to the characteristics of neoplastic cells

Chemotherapeutic treatment	Cancer characteristics	Effects
	Active p53-pRb pathway with High ribosome biogenesis rate	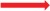 Apoptosis
Drugs inhibiting rRNA synthesis		
e.g: Doxorubicin, 55FU, Methotrexate, Oxaliplatin Actinomycin D, CX 5461 etc.	Active p53-pRb pathway with Low ribosome biogenesis rate	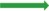 Cell cycle arrest
	Inactivated p53-pRb pathway (Mutated TP53 and RB loss or pRb hyperphosphorylation)	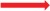 Apoptosis
